# Foreign Body in the Larynx—Laryngotomy

**Published:** 1844-08

**Authors:** C. J. Thornton

**Affiliations:** Henderson, Ky.


Art. IV.—*Foreign body in the Larynx—Laryngotomy.* By C. J. Thornton, M.D., of Henderson, Ky.

The subject of this case, a healthy male child, twenty-one months old, was playing in the garden with beans of the long white variety, when suddenly the attention of his mother was attracted by fits of coughing which seemed to deprive him of breath. After successive paroxysms, he became comparatively quiet. At intervals, however, he was attacked as at first, but not so violently. I was consulted about three weeks after the accident occurred, and upon examination readily detected the presence of a foreign body in the larynx, passing to and from the trachea with every inspiration and expiration. As yet but little inflammation existed, less, indeed, than might have been expected; but from the small probability of foreign bodies being expelled from this location, I advised an operation at once. The parents not consenting, I was compelled to wait the course of events.

Some weeks after, I was hastily summoned, and now found the child's condition truly alarming. There was laborious breathing, with stridor; extensive inflammation of the mucous membrane; constant and distressing cough, and puriform expectoration.

Assisted by Dr. Read, in the presence of Drs. Swan and

Boram, I commenced the operation by an incision through the skin and cellular substance, proceeding rapidly, but carefully to divide the crico-thyroid membrane. It was necessary to secure two branches of the thyroid artery, and a branch of the inferior thyroid vein which bled profusely. After extending the incision as far as practicable, I found it impossible to dilate the blades of the forceps sufficiently to seize the bean, and on a moment's reflection, I determined to make an effort to force it with a bullet-pointed probe through the rima glottidis, seeking an opportunity to take it, as it were, by surprise. This I readily effected, owing in a great measure to the smooth and rounded surface of the bean.

This mode of effecting the escape of a rough or irregular body, would be difficult, if not impracticable. I see that Sir B. C. Brodie lately succeeded in a similar attempt; though it would appear from the experiments of Mr. Erichsen (Lancet, 1843) that the irritation and consequent spasmodic constriction of the glottis, would, under all circumstances, render this effort at the expulsion of foreign bodies abortive. I will only add, that the child recovered speedily, and is at this time well.

Henderson, July 5, 1844.

